# Relationships between mothers and children in families formed by shared biological motherhood

**DOI:** 10.1093/humrep/dead047

**Published:** 2023-03-09

**Authors:** Susan Golombok, Kate Shaw, Anja McConnachie, Vasanti Jadva, Sarah Foley, Nick Macklon, Kamal Ahuja

**Affiliations:** Centre for Family Research, University of Cambridge, Cambridge, UK; Centre for Family Research, University of Cambridge, Cambridge, UK; Centre for Family Research, University of Cambridge, Cambridge, UK; Institute for Women’s Health, University College London, London, UK; Moray House School of Education and Sport, University of Edinburgh, Edinburgh, UK; London Women’s Clinic, London, UK; London Women’s Clinic, London, UK

**Keywords:** lesbian mothers, shared motherhood, mother–child relationships, bonding, donor-IVF, Parent Development Interview

## Abstract

**STUDY QUESTION:**

Does shared biological motherhood, in which a woman gives birth to the genetic child of her female partner, result in more positive mother–child relationships than donor insemination, in which only one mother is biologically related to the child?

**SUMMARY ANSWER:**

Mothers in both family types showed high levels of bonding with their children and viewed their relationship with their child positively.

**WHAT IS KNOWN ALREADY:**

There is some evidence of feelings of inequality regarding their relationship with their child between biological and non-biological mothers in lesbian mother families formed by donor insemination, with a qualitative longitudinal study showing a tendency for children to form stronger bonds with their biological than their non-biological mother.

**STUDY DESIGN, SIZE, DURATION:**

Thirty lesbian mother families created through shared biological motherhood were compared with 30 lesbian mother families formed by donor-IVF. All families had two mothers who both participated in the study, and the children were aged from infancy up to 8 years old. Data collection took place over 20 months beginning in December 2019.

**PARTICIPANTS/MATERIALS, SETTING, METHODS:**

Each mother in the family was interviewed separately using the Parent Development Interview (PDI), a reliable and valid measure of the nature of the parent’s emotional bond with their child. The interviews were transcribed verbatim and coded separately by one of two trained researchers who were unaware of the child’s family type. The interview produces 13 variables that relate to the parent’s representations of themselves as a parent, 5 variables that relate to the parent’s representations of the child, and a global variable that assesses the extent to which the parent can reflect on the child and their relationship.

**MAIN RESULTS AND THE ROLE OF CHANCE:**

Families formed through shared biological parenthood did not differ from families created by donor-IVF in terms of the quality of mothers’ relationships with their children as assessed by the PDI. Neither were differences identified between birth mothers and non-birth mothers across the entire sample, or between gestational and genetic mothers within the families formed by shared biological parenthood. Multivariate analyses were conducted to minimize the role of chance.

**LIMITATIONS, REASONS FOR CAUTION:**

Ideally, larger samples of families and a narrower age range of children would have been studied, but this was not possible as we were reliant on the small number of families formed through shared biological motherhood in the UK when the study began. To maintain the anonymity of the families, it was not possible to request information from the clinic that may have shed light on differences between those who responded to the request to participate and those who did not.

**WIDER IMPLICATIONS OF THE FINDINGS:**

The findings show that shared biological motherhood is a positive option for lesbian couples who wish to have a more equal biological relationship to their children. One type of biological connection does not appear to have a greater influence on the quality of parent–child relationships than the other.

**STUDY FUNDING/COMPETING INTEREST(S):**

This study was funded by the Economic and Social Research Council (ESRC) grant ES/S001611/1. KA is Director, and NM is Medical Director, of the London Women’s Clinic. The remaining authors have no conflicts of interest to declare.

**TRIAL REGISTRATION NUMBER:**

N/A.

## Introduction

The growth of assisted reproduction in the 1980s resulted in donor insemination becoming a new route to parenthood for lesbian women. For the first time, rather than fighting for custody of their children following an acrimonious divorce, lesbian couples began to plan their family together after coming out. The rapid increase in lesbian women having children at that time became known as ‘the lesbian baby boom’ (Patterson, 1995). In lesbian mother families formed through donor insemination, the mother who becomes pregnant has a biological connection to the child whereas the other mother does not. Throughout the article, the term ‘biological’ refers to the combination of gestational and genetic parenthood, whereas the terms ‘gestational’ and ‘genetic’ refer to these specific aspects of biological parenthood. The terms ‘gestational mother’ and ‘birth mother’ are used interchangeably. A number of studies of planned lesbian mother families with children born through donor insemination have been carried out, showing no differences in the quality of parent–child relationships or children’s psychological adjustment between these families and demographically matched comparison groups of heterosexual parent families also formed through donor insemination (Brewaeys *et al.*, 1997; Golombok *et al.*, 1997; Chan *et al.*, 1998; Bos *et al.*, 2004, 2007; MacCallum and Golombok, 2004; Bos and Van Balen, 2008; Golombok and Badger, 2010).

Investigations of parenting in lesbian mother families have found lesbian mothers to share parenting more equally than do heterosexual parents (Patterson, 2013; Patterson *et al.*, 2014). However, a qualitative longitudinal study designed to produce in-depth data on the dynamics of lesbian mother families with donor-conceived children found that, in the preschool years, biological mothers were more likely to be the primary caregiver, and two-thirds of the mothers acknowledged some jealousy and competitiveness regarding bonding with the child (Gartrell *et al.*, 1999). When the children reached 5 years, one-third of the couples thought that the child was more securely attached to the biological mother, with the remaining two-thirds reporting that the child was equally attached to both mothers (Gartrell *et al.*, 2000). By age 10, half of the couples reported that the child showed a stronger bond to the biological mother, and some non-biological mothers continued to experience jealousy or competitiveness regarding their relationship with the child (Gartrell *et al.*, 2006). Thus, it appeared that there was a tendency for children to form more secure attachments to their biological than their non-biological mother. In an ethnographic study, Pelka (2009) similarly reported feelings of jealousy and being excluded by non-biological mothers in families with children born through donor insemination, Wojnar and Katzenmeyer (2014) found that non-biological mothers felt different from preconception until after the baby was born, and McKelvey (2014) highlighted the role of breastfeeding in creating a difference in the relationship between the biological and non-biological mother and the child.

In 2010, lesbian couples began to have children through ROPA (Reception of Oocytes from PArtner) treatment whereby one woman’s egg is used to create an embryo with donated sperm and the other woman carries the pregnancy (Marina *et al.*, 2010; Yeshua *et al.*, 2015; Carpinello *et al.*, 2016). This procedure enables both mothers to have a biological connection to their child; the mother who provides the egg has a genetic connection and the mother who hosts the pregnancy has a gestational connection. Today, the term shared biological motherhood is more commonly used to refer to this procedure, although it is also referred to as reciprocal IVF and intra-partner egg donation. In a study of 121 couples, Bodri *et al.* (2018) found shared biological motherhood to be a highly successful and safe treatment for lesbian women.

The present study addresses the question of whether shared biological motherhood, in which both mothers have a biological connection to the child, results in more positive mother–child relationships than donor insemination, in which only one mother is biologically related to the child. Couples who opt for shared biological motherhood do so not only because they wish to be more equal in their relationship to their child but also because they wish to be viewed more equally by others (Pelka, 2009). Greater equality in their biological relatedness to their child may ameliorate the feelings of competitiveness and jealousy that have been reported by lesbian mothers in families formed by donor insemination, and may result in more positive family functioning.

Research on lesbian mother families formed through shared biological motherhood is of interest in its own right as it provides empirical data on the outcomes for parents and children in families formed in this novel way. However, this research is also of broader theoretical interest as, by separating aspects of parenting that usually go together, it can increase understanding of the role of gestational, genetic, and social connections in parenting, more generally. Comparisons between birth mothers and non-birth mothers enable the examination of the role of pregnancy and birth in the quality of birth mothers’ relationships with their children. Due to the growing body of research showing that feelings of bonding between a mother and her baby begin to develop during pregnancy and influence the nature of the mother–child relationship after the baby is born (Walsh *et al.*, 2013; Foley and Hughes, 2018), it was hypothesized that the relationship between birth mothers and their children would be more positive than those of non-birth mothers and their children. Although there is evidence that fathers in heterosexual parent families also start to bond with their babies prenatally (de Cock *et al*., 2016; Foley and Hughes, 2018), which suggests that the same would be true of lesbian mothers who do not give birth to their child, the phenomenon of prenatal bonding is generally assumed to be enhanced by the experience of pregnancy (Glover and Capron, 2017). Breastfeeding has also been associated with maternal bonding (Farrow and Blissett, 2014), which would again lead to the expectation that the gestational mothers, should they choose to breastfeed, would develop a stronger relationship with the child.

Families formed through shared biological motherhood also allow, for the first time, a direct comparison between the quality of children’s relationships with a genetic and gestational mother within the same family, thus avoiding the potentially confounding effects of between-family differences, and enabling the examination of whether a genetic or a gestational connection is more important for maternal bonding. For the reasons outlined above regarding the role of pregnancy in promoting bonding with the child, the third hypothesis was that gestational mothers would experience a more positive relationship with the child than genetic mothers within families formed through shared biological parenthood.

## Materials and methods

Data on parent–child relationships were collected by audio-recorded, semi-structured, standardized interviews with each parent separately over a period of 20 months beginning in December 2019. Due to Covid restrictions, almost all of the interviews were carried out online. The interview transcripts were coded by two researchers trained in the coding scheme for the interview who had not conducted the interviews themselves.

### Participants

Thirty lesbian mother families created through shared biological motherhood were compared with 30 lesbian mother families formed by donor IVF, to provide a comparison group of families in which only one mother had a biological connection to the child. As the families in the comparison group had conceived their child using IVF, rather than donor insemination alone, this controlled for the demands of IVF experienced by the shared parenting group.

The families were recruited through the London Women’s Clinic, the first clinic to introduce shared biological motherhood to the UK in 2011. To maintain confidentiality, the families were initially approached by the clinic to request permission for the researchers to contact them to provide further information about the study. The inclusion criteria for the study were that the families were headed by two lesbian mothers who had raised their child together in the family home, and that the children were aged up to 8 years old. One hundred and twelve couples with a child born through shared biological motherhood, and 179 couples with a child born through donor IVF, were contacted by the clinic by email. Forty-seven shared biological motherhood families and 74 donor IVF families responded, representing 42% and 41% of each family type, respectively.

Of those who responded, 87% of the shared biological parenthood families and 93% of the donor IVF families met the inclusion criterion of having a child aged 8 years old or younger. The two groups of families were matched as closely as possible for the ages of the children to maximize comparability on this key variable, followed by other demographic characteristics such as the gender of the children, the age of the mothers, the number of siblings in the household, and the presence of financial difficulties.

The present paper is based on 30 shared biological motherhood families and 30 donor IVF families in which both mothers participated in the study and the mothers identified as cis-gender. Due to delays caused by Covid, it was decided to terminate recruitment when 30 of such families in each family type had taken part in the study. Findings on mothers’ motivations for, and experiences of, shared biological motherhood for the larger sample are reported elsewhere (Shaw *et al*., 2023).

Univariate ANOVAs found no significant differences between family types in either the age of the birth mothers (*F* = 3.00, *P* = 0.08, *d*^1^ = 0.44), the non-birth mothers (*F* = 3.73, *P* = 0.06, *d*^1^ = 0.49), or the children (*F* = 3.69, *P* = 0.06, *d*^1^ = 0.49). Similarly, Chi-square tests showed no significant differences between the shared biological parenthood families and donor IVF families in either the gender of the children (χ^2^ = 0.27, *P* = 0.60), the number of siblings in the household (χ^2^ = 1.79, *P* = 0.40), or the presence of financial difficulties (χ^2^ = 2.96, *P* = 0.08).

### Measures

#### Mothers’ perceptions of relative closeness to child, jealousy, and feeding arrangements

In terms of the child’s relative closeness to each mother, birth mothers and non-birth mothers were asked separately to endorse one of the following statements: child is closer to birth mother, child is equally close to both mothers, or child is closer to non-birth mother. In addition, birth mothers and non-birth mothers were asked to endorse one of the following statements regarding jealousy towards their partner: no jealousy experienced, a little jealousy experienced, or considerable jealousy experienced. Regarding feeding arrangements, each family was categorized according to whether the birth mother breastfed the baby exclusively, or the couple shared feeding using a combination of breast and bottle feeding.

#### Parent development interview

Each mother in the family was interviewed separately using the Parent Development Interview [PDI] (Slade *et al.*, 1999; Slade 2005; Henderson *et al.*, 2007), a reliable and valid measure of the nature of the parent’s emotional bond with their child. The PDI is designed to assess parents’ representations of their child, themselves as parents, and their relationship with their child. Parents are asked to describe their experiences, and their child’s experiences, in moments of relatedness and interaction to provide a window into their own, and their child’s, thoughts and feelings. The PDIs were transcribed verbatim and coded by one of two trained researchers who were unaware of the child’s family type. The PDI produces 13 Parent Affective Experience variables, which relate to the parents’ representations of themselves as a parent; 5 Child Affective Experience variables, which relate to the parents’ representations of the child; and a global code of Reflective Functioning. Each variable was rated on a four-point scale, with higher scores representing a higher level of the construct.

The variables relating to the affective experience of the parent are as follows: (i) *degree of anger* assesses the extent to which the parent feels angry in the relationship with their child; (ii) *expression of anger* measures the extent to which expressions of anger are present in the parent’s relationship with their child; (iii) *support need* measures the parent’s acknowledgment of their need for support; (iv) *support satisfaction* assesses parental satisfaction with the level of support available to them; (v) *guilt* assesses the degree to which parental guilt is a feature of the parent–child relationship; (vi) *joy* measures the parent’s ability to express feelings of joy, contentment and happiness in their relationship with the child; (vii) *competence* assesses how well the parent is coping in parenting the child; (viii) *confidence* measures the parent’s view of their own competence; (ix) *child focus* assesses the degree to which the parent is focused on the needs of the child as compared with their own emotional needs; (x) *disappointment* measures the extent to which the parent expresses disappointment in the role of being a parent; (xi) *warmth* assesses the amount of warmth the parent feels towards their child; (xii) *attachment awareness* measures the parent’s understanding of attachment issues for their child and their ability to behave in ways that will promote the child’s attachment to them; and (xiii) *hostility* assesses the parent’s hostile feelings towards the child. After reverse coding the negatively worded items, a mean aggregate parent affective experience score was computed, whereby a higher score reflected a more positive experience (alpha = 0.86).

The following variables relate to the parent’s representations of the child: (i) *child anger* assesses the degree to which the parent represents the child as experiencing or expressing anger; (ii) *child happiness* assesses the degree to which the parent represents the child as happy and contented within themselves as distinct from the parent–child relationship; (iii) *child controlling* measures the extent to which the child attempts to both control the parent and interactions more generally; (iv) *child affection* measures the extent to which the child shows and accepts physical affection in relation to their parents; and (v) *child rejection* assesses the degree to which the parent feels emotionally or practically rejected by the child. After reverse coding the negatively worded items, a mean aggregate child affective experience score was computed, whereby a higher score reflected a more positive experience (alpha = 0.68).

The global code, reflective functioning, assesses the extent to which the parent can reflect upon the child and their relationship. Parents who obtain a high score show deep and prolonged thinking about the nature of the relationship and the influences upon it, and a considerable amount of empathy, understanding and sensitivity towards the child, whereas parents with a low score are reluctant to accept that they have a substantial role in shaping their child’s life, and tend to believe that the child’s difficulties are the responsibility of the child alone.

To establish the inter-rater reliability of the PDI variables, interview transcripts from a random selection of one-third of the families were re-coded independently by the other coder and Pearson correlation coefficients calculated for each variable.

### Analysis plan

To account for the dyadic nature of the data from the PDI (Kenny *et al.*, 2006), i.e. that data were obtained from both partners in the couple, multilevel models (MLM) were conducted to test the research questions relating to parent affective experience, child affective experience and reflective functioning. Two-level random intercept models were tested to estimate the amount of variation in each of these three outcome variables that was accounted for by family- and parent-level factors. The analyses were carried out in four stages. The first baseline model (Model 1) estimated the overall couple-level variance for each outcome variable. The second model related to the first research question of whether shared biological motherhood resulted in more positive mother–child relationships than donor IVF. It included Level 2 measures to examine variance occurring between family types, specifically, a variable to reflect whether the family type was formed through shared biological parenthood or donor IVF, and the age of the child as a covariate. The third model addressed the second research question of whether mothers who gave birth had more positive relationships with their children than non-birth mothers. Model 3 included Level 1 measures to examine variation in the outcome variables accounted for by variance occurring within the families, specifically, a variable to represent whether the mother was a birth mother or a non-birth mother, and a variable to represent whether she was a genetic or non-genetic mother, and the age of the mother as a covariate. The fourth model tested whether the findings held when both Level 1 and Level 2 measures were entered simultaneously, and parent age and child age were entered as covariates. An interaction was added to this model to address the third research question of whether, within the shared biological parenthood families, the gestational mothers experienced more positive relationships with their children than the genetic mothers. This model included both Level 1 and Level 2 measures simultaneously, and the interaction between family type and parent type to explore the difference between the gestational and genetic mothers within the shared biological parenthood families.

MLM analyses were conducted in M*plus* version 8 (Muthén and Muthén, 1998–2017) using maximum likelihood estimation to estimate model parameters and standard errors. At each stage, changes in model-fit were estimated to assess whether the addition of new predictors explained significantly more variation in the three outcome variables. The intra-class correlation coefficient (ICC) was used to examine the correlation between mothers in the same family (i.e. the couple), with a higher number suggesting that the observations were not independent (Hox, 2010 suggested that 0.10, 0.20, and 0.30 reflect small, medium, and large values, respectively), and changes between each model provided an indication of the amount of variance the new measures explained. Based on simulation work (Du and Wang, 2016), our sample of 60 couples (with no missing data) exceeds the suggested minimum of 50 couples with no missing data needed to obtain reliable and valid estimates.

## Results

### Mothers’ perceptions of relative closeness to child, jealousy, and feeding arrangements

As shown in Table I, in terms of the child’s relative closeness to each mother, there was no difference between family types based on the accounts of either birth mothers (χ^2^ = 1.20, *P* = 0.54), or non-birth mothers (χ^2^ = 3.09, *P* = 0.21). The majority of children were equally close to both mothers, most of the remaining children were closer to the birth mother, and a small minority of children were closer to the non-birth mother. In addition, there were no differences in feelings of jealousy towards their partner between birth mothers (χ^2^ = 1.41, *P* = 0.49), or non-birth mothers (χ^2^ = 2.69, *P* = 0.26), with most mothers experiencing no feelings of jealousy, around one-third experiencing a little jealousy, and less than 10% feeling considerable jealousy. Neither was there a difference between family types in feeding (χ^2^ = 0.07, *P* = 0.5). Most couples chose a combination of breast and bottle feeding, with around 40% of birth mothers in both family types breastfeeding their babies exclusively.

**Table I:** Means, SD, χ^2^, and *P* values for mothers’ perceptions of relative closeness to child, jealousy, and feeding arrangements by family type.

	Shared biological motherhood		Donor IVF			
	N	%	N	%	χ^2^	*P*
Birth mother rating of child closeness						
Closer to birth mother	9	30%	11	37%	1.20	0.54
Equally close	18	60%	18	60%		
Closer to non-birth mother	3	10%	1	3%		
Non-birth mother rating of child closeness						
Closer to birth mother	10	33%	13	43%	3.09	0.21
Equally close	15	50%	16	53%		
Closer to non-birth mother	5	17%	1	4%		
Birth mother experienced jealousy						
No	16	62%	20	67%	1.14	0.49
A little	7	27%	9	30%		
Considerable	3	11%	1	3%		
Non-birth mother experienced jealousy						
No	15	52%	20	72%	2.69	0.26
A little	12	41%	6	21%		
Considerable	2	7%	2	7%		
Feeding						
Breastfeeding only	12	40%	13	43%	0.07	0.50
Breast and bottle feeding	18	60%	17	57%		

### Parent Development Interview

#### Inter-rater reliability

In terms of the inter-rater reliability of the PDI, Pearson correlation coefficients ranged from 0.86 to 0.96 for the Parent Affective Experience variables, from 0.82 to 0.96 for the Child Affective Experience variables, and the correlation coefficient for Reflective Functioning was 0.88. Descriptive statistics for the PDI variables are illustrated in Table II.

**Table II:** Descriptive statistics for PDI ratings of parent affective experience, child affective experience, and parental reflective functioning.

PDI rating	Mean	SD	Range
Parent affective experience			
Degree of anger	2.20	0.55	1–3.5
Expression of anger	1.83	0.64	1–3
Support need	2.00	0.35	1–3
Support satisfaction	3.65	0.59	1.5–4
Guilt	2.07	0.62	1–4
Joy	3.52	0.56	2–4
Competence	3.00	0.56	1.5–4
Confidence	3.06	0.52	2–4
Child focus	3.39	0.63	2–4
Disappointment	1.27	0.42	1–2.5
Warmth	3.80	0.41	1.5–4
Attachment awareness	3.47	0.64	2–4.5
Hostility	1.08	0.27	1–2
Child affective experience			
Child anger	2.10	0.52	1–3
Child happiness	3.43	0.56	1–4
Child controlling	1.67	0.58	1–3
Child affection	3.56	0.59	1.5–4
Child rejection	1.51	0.57	1–3
Global code			
Parent reflective functioning	3.33	0.61	1.5–4

PDI, Parent Development Interview.

#### Parent affective experience

As illustrated in Table III, the baseline model for parent affective experience indicated that a moderate proportion of variance was accounted for by the couple. Family-level factors were added in Model 2 and did not significantly improve the fit of the model (log likelihood test, Δ(2) = 3.27, *P *>* *0.05 and non-significant differences in the ICC), indicating that family type, i.e. shared biological parenthood versus donor IVF, was not associated with differences in parent affective experience. Including only parent-level factors in Model 3 did not significantly improve the fit of the model compared to the baseline (log likelihood test, Δ(4) = 1.64, *P* > 0.05 and non-significant differences in the ICC), indicating that there was no difference in parent affective experience between the mothers who gave birth and those who did not. These findings did not differ when both family-level and parent-level factors were simultaneously entered into Model 4. Finally, the cross-level interaction between family type and type of parenthood was non-significant (Estimate (SE) = −0.01 (0.09), *P *=* *0.898), indicating that there was no difference between gestational and genetic mothers within the shared biological parenthood families.

**Table III:** Results of multilevel model examining associations between parent and family characteristics and PDI ratings of parent affective experience, child affective experience, and parental reflective functioning.

Level predictors	Model 1 baseline			Model 2 family-level predictors			Model 3 parent-level predictors			Model 4 family and parent level predictors		
	Estimates (SE)			Estimates (SE)			Estimates (SE)			Estimates (SE)		
	Parent affect	Child affect	Parent RF	Parent affect	Child affect	Parent RF	Parent affect	Child affect	Parent RF	Parent affect	Child affect	Parent RF
Parent level												
Genetic	–	–	–	–	–	–	0.02 (0.04)	0.01 (0.06)	0.00 (0.09)	0.02 (0.04)	0.01 (0.06)	0.00 (0.10)
Birth	–	–	–	–	–	–	0.01 (0.04)	0.07 (0.06)	0.15 (0.09)	0.01 (0.04)	0.07 (0.06)	0.15 (0.10)
Genetic Parent Age	–	–	–	–	–	–	0.10* (0.01)	−0.02 (0.03)	0.10 (0.11)	0.10 (0.01)	−0.02 (0.03)	0.10 (0.11)
Birth Parent Age	–	–	–	–	–	–	0.03 (0.02)	−0.03 (0.03)	−0.02 (0.12)	0.03 (0.02)	−0.03 (0.02)	−0.02 (0.12)
Family level												
Family type	–	–	–	−0.09 (0.07)	−0.01 (0.08)	−0.23 (0.12)	–	–	–	−0.09 (0.07)	−0.01 (0.08)	−0.23 (0.02)
Child age	–	–	–	−0.04 (0.02)	−0.10 (0.02)	0.02 (0.03)	–	–	–	−0.04 (0.01)	−0.01 (0.02)	0.02 (0.03)
ICC	0.44	0.31	0.31	0.44	0.31	0.31	0.45	0.32	0.34	0.45	0.32	0.34
Intercept	3.32 (0.04)	3.34 (0.04)	3.33 (0.06)	3.51 (0.07)	3.38 (0.08)	3.38 (0.08)	3.31 (0.05)	3.30 (0.06)	3.26 (0.10)	3.50 (0.08)	3.34 (0.10)	3.30 (0.14)

PDI, Parent Development Interview; Parent RF, parental reflective functioning; parent affect, parent affective experience; child affect, child affective experience; Genetic, genetic relationship to child; birth, birth parent.

* *P* < 0.05.

#### Child affective experience

The baseline model for child affective experience indicated that a moderate proportion of variance was accounted for by the couple (see Table III). Family-level factors were added in Model 2 and did not significantly improve the fit of the model (log likelihood test, Δ(2) = 0.12, *P* > 0.05 and non-significant differences in the ICC), showing that family type was not associated with differences in child affective experience. Model 3 tested parent-level factors only, and did not significantly improve the fit of the model (log likelihood test, Δ(4) = 0.85, *P* > 0.05 and non-significant differences in the ICC), indicating that the mothers who gave birth did not differ from those who did not in terms of child affective experience. These findings did not differ when both family-level and parent-level factors were simultaneously entered into Model 4. The cross-level interaction between family type and type of parenthood was non-significant, Estimate (SE) = 0.06 (0.10), *P *=* *0.308, showing that there was no difference in child affective experience between gestational and genetic mothers within the shared biological parenthood families.

#### Reflective functioning

The baseline model for reflective functioning also indicated a moderate proportion of variance was accounted for by the couple (see Table III). Again, the addition of family-level factors in Model 2 did not significantly improve the fit of the model (log likelihood test, Δ(2) = 2.27, *P *>* *0.05 and non-significant differences in the ICC), showing that there was no difference between the shared biological parenthood families and the donor IVF families with respect to reflective functioning. Model 3 included the parent-level factors only and did not significantly improve the fit of the model compared to the baseline (log likelihood test, Δ(4) = 2.13, *P *>* *0.05 and non-significant differences in the ICC), indicating that there was no difference in reflective functioning between the birth mothers and the non-birth mothers. These findings did not differ when both family-level and parent-level factors were simultaneously entered into Model 4. The cross-level interaction between family type and type of parenthood was non-significant, Estimate (SE) = 0.30 (0.18), *P *=* *0.101, showing no difference in reflective functioning between the gestational and genetic mothers within the shared biological parenthood families.

## Discussion

Families formed by shared biological parenthood did not differ from families created through donor IVF in terms of the quality of mothers’ relationships with their children as assessed by the parent affective experience, child affective experience, and reflective functioning scales of the PDI. Mothers in both family types showed high levels of bonding with their children and viewed their relationship with their child positively, with their scores on the PDI reflecting high levels of joy, warmth, competence, and confidence in their role as a parent, and low levels of anger and hostility towards their child, as well as little disappointment in being a parent. They also viewed their child as happy and affectionate, rather than rejecting, and showed the capacity to make sense of their child’s internal experiences as well as their own experiences as a parent, both of which are associated with sensitive parenting and children’s psychological adjustment (Slade, 2005; Fearon and Roisman, 2017; Oppenheim and Koren-Karie, 2021).

It seems, therefore, that the couples’ choice of either shared biological parenthood, where both mothers have a biological connection to the child, or donor IVF, where only one mother has a biological connection to the child, resulted in similarly positive family relationships. It may be relevant that there were no differences in mothers’ perceptions of closeness to the child, or feelings of jealousy regarding their partner’s relationship with the child, between the family types. Moreover, a similar proportion of couples in each family type participated in the feeding of their baby.

Neither were differences identified in the parent affective experience variables, the child affective experience variables, or reflective functioning between birth mothers and non-birth mothers. Contrary to the hypothesis that birth mothers would have closer relationships with their children than non-birth mothers, this finding suggests that the mother’s involvement with the child is more important for bonding than the experience of pregnancy and birth. This finding is in line with attachment theory in that variation in attachment is considered to be socially, rather than biologically, determined, with sensitive responding by parents as the main social influence on their children’s attachment security (Bowlby, 1969; Ainsworth, 1985; Fearon and Roisman, 2017). Moreover, a central tenet of attachment theory is that children may form attachments to more than one parent, and that the child’s primary attachment figure need not be the birth parent (Bowlby, 1988).

The absence of differences between birth and non-birth mothers is also consistent with the findings of studies of children adopted in infancy, which show no differences in mother–child attachment between adoptive and non-adoptive families (Palacios and Brodzinsky, 2010), and also with studies of children born through surrogacy, also raised by mothers who have not given birth to them, who show similarly positive relationships with their mothers as children born through unassisted conception (Golombok *et al.*, 2004a, 2006a,b, 2011a, 2017). Thus, the findings of the present study add to the increasing body of evidence that a gestational connection between a mother and her child is not essential for a positive relationship to develop between them (Golombok, 2020; Imrie and Golombok, 2020).

Regarding the relative importance of a gestational or a genetic connection to the child for maternal bonding, there were no differences between the gestational and genetic mothers in families formed by shared biological parenthood for any of the parent or child affective experience variables, or reflective functioning, indicating that one type of biological relatedness did not have a greater influence than the other on the quality of mother–child relationships. Thus, the prediction that the gestational mother would have a closer relationship with the child than the genetic mother in families created through shared biological parenthood lacked empirical support. This finding is consistent with the similarly positive mother–child relationships found in studies of families formed by surrogacy (Golombok *et al.*, 2004a, 2006a,b, 2011a, 2017), as noted above, and egg donation (Golombok *et al.*, 2004b, 2005, 2006b, 2011b, 2017) where mothers are genetically unrelated to their children. Again, it appears that the mother’s involvement in rearing the child is more influential in the development of a positive mother–child relationship than the presence of a gestational or a genetic connection between them.

It is important to emphasize that both family types were functioning well, with their scores on the interview variables reflecting positive representations of parenthood, their child, and their relationship with their child. The mothers’ scores were closely comparable to the scores of heterosexual mothers with children born through unassisted conception (Golombok *et al.*, 2006), and more positive than the scores of mothers of children adopted beyond infancy (Steele *et al.*, 2008; Steele and Steele, personal communication). Although the interview was designed to assess parents’ bonding to their children, as opposed to children’s attachment to their parents, and children’s attachment relationships were not directly assessed, the PDI is based on a large body of theory and research showing that a parent’s thoughts and feelings about their child influences their parenting behaviour (Slade *et al.*, 1999; Slade 2005; Oppenheim and Koren-Karie, 2021). Thus, the children in the present study would be expected to show positive outcomes in terms of the security of their attachment relationships with their mothers and psychological adjustment.

A particular advantage of the study was the use of an in-depth, theoretically based and psychometrically sound measure of the nature and quality of a parent’s relationship with their child. A limitation of the study was that, for both family types, the sample sizes for which data were obtained from both parents in each family, were modest. Moreover, the high levels of inter-rater reliability that were demonstrated with the present sample meant that only moderate to large differences between them were likely to have been detected. At an alpha of 0.05, this sample provided 80% power to detect large differences between the two groups, and 60% power to detect medium differences (Cohen, 1992; Faul *et al.*, 2007). Nevertheless, the sample size of 60 couples with no missing data was greater than the suggested minimum of 50 couples required to obtain reliable and valid estimates of group differences in multi-level modelling. Moreover, this is the first quantitative study of this emerging family form, and although the findings should be interpreted tentatively, they provide preliminary evidence that there are no marked adverse effects of shared biological parenthood on family relationships.

Ideally, a narrower age range of children would have been studied, but this was not possible as we were reliant on the small number of families formed through shared biological motherhood in the UK when the study began. As the number of such families has increased over time, future research will be able to focus on more specific stages of children’s development, for example, by differentiating between the breastfeeding and post-breastfeeding years, and establish whether the findings of this initial study are replicated with larger samples of families with children in more focused age groups. The participation rate in the study was high among those couples who agreed to be contacted by the researchers. In order to maintain the anonymity of the families, it was not possible to request information from the clinic that may have shed light on differences between those who responded to the request to participate and those who did not. A further issue relates to selection bias. As it is not possible to randomly allocate couples to shared biological motherhood or donor IVF, the findings are confounded by the couples’ choice of one or other route to parenthood. However, the decision to opt for shared biological motherhood or donor IVF is an intrinsic characteristic of the samples studied, and thus the samples are typical of families created in these ways.

Overall, the findings of this study show that shared biological motherhood appears to be a positive option for lesbian couples who wish to have a more equal biological relationship to their children. The findings not only have implications for lesbian couples, but they also increase understanding of the relative importance of genetic and gestational relatedness for the quality of parent–child relationships, more generally. One type of biological connection does not appear to have a greater influence than the other on the quality of parent–child relationships. Instead, it is the psychosocial relationship between parents and their children that appears to have a dominant effect.

## Data Availability

The data underlying this article cannot be shared to protect the anonymity of the families.
